# Distortion of the Face Cured by Disease of the Brain

**Published:** 1830-01-01

**Authors:** 


### XLVIII.

### DISTORTION OF THE FACE CURED BY DISEASE OF THE BRAIN.

Thanks to the ingenuity and perseverance of Mr. Charles Bell, the twisting of the side of the face produced by paralysis of the portio dura, depending on local causes, is now pretty generally understood. The distortion which this partial palsy produces is sometimes very frightful and always very odd, but we fancy that few patients, notwithstanding, would chuse to be cured in the following manner.

"*Case.* A man, aged 36, about a year before his death, had a tumor extirpated from behind the angle of the jaw, on the left side, and immediately after the operation, paralysis took place in the left side of the face, in consequence of which his mouth was distorted to the opposite side in a most extraordinary degree. About six months after this, he began to complain of headach, and giddiness, which often gave him the appearance of intoxication, and after some time these symptoms were followed by impaired vision, occasional strabismus, and a considerable degree of deafness; and at last by drowsiness, coma, convulsions, and death. As these symptoms advanced, he became affected with numbness, and loss of power of the right side of the face, which increased very gradually. During the increase of this, the distortion of his mouth gradually diminished, and for some time before his death, his countenance had become entirely symmetrical. Both sides of his face were now entirely paralytic, but with this difference, that on the right side, the feeling was also lost, while on the left the feeling was entire.

"*Inspection.* In the centre of the middle lobe of the right hemisphere of the brain, there was a tubercle about an inch long, and three-fourths of an inch in breadth. At its lower part it was attached to the cerebral substance, but the rest of it was detached, being surrounded with dark-coloured pus. In the vicinity, there was increased vascularity with softening of the cerebral substance."—*Abercrombie. 2d Edition.*

By the way, whilst writing of paralysis of the face, we may take occasion to remark the confusion into which authors have fallen, respecting the side on which this occurs in hemiplegia. Some have stated that the face and the body are affected on the same side, others the contrary. The fact is, that much variation exists in this respect, but we would be disposed to say, that the face and the body are most commonly paralysed on opposite sides. Thus, if disease exist in the left hemisphere of the brain, the patient will generally be hemiplegiac on the right side, but the portio dura will be palsied on the same side as the disease in the brain, and, consequently, not on the same as the body.

